# A geospatial inventory of regulatory information for wine protected designations of origin in Europe

**DOI:** 10.1038/s41597-022-01513-0

**Published:** 2022-07-11

**Authors:** Sebastian Candiago, Simon Tscholl, Leonardo Bassani, Helder Fraga, Lukas Egarter Vigl

**Affiliations:** 1 Institute for Alpine Environment, Eurac Research, Viale Druso 1, 39100 Bozen/Bolzano, Italy; 2 Ca’ Foscari University of Venice, Department of Economics, S. Giobbe 873, 30121 Venezia, Italy; 3 Department of Ecology, University of Innsbruck, Innrain 52, 6020 Innsbruck, Austria; 4 Centre for the Research and Technology of Agro-Environmental and Biological Sciences, University of Trás-os-Montes and Alto Douro, 5000-801 Vila Real, Portugal

**Keywords:** Agriculture, Environmental social sciences

## Abstract

The Wine Protected Designation of Origin (PDO) label is a European quality scheme that protects high quality wines by linking them to legally defined geographic areas and a set of specific production practices. Because of the tight relation between PDO wines and the specifications defined in the official regulatory documents, these products are highly susceptible to changes in climatic, environmental, or socioeconomic conditions. However, the content of these regulatory documents has never been systematically analysed and summarized in a single dataset. Here, we present the first geospatial inventory that organizes regulatory information about the 1177 wine PDO in Europe based on the documents from the official EU geographical indication register. It includes essential legal information that defines the wine PDO such as the geographic boundaries, authorized cultivars and maximum yields. This inventory opens new possibilities for researchers to accurately assess, compare and map the regulatory information in each wine region at an unprecedented level of detail, supporting decision makers in developing adaptation strategies for the preservation of PDO wine regions.

## Abstract (summary)

**Table:** 

Measurement(s)	Wine • Agriculture
Technology Type(s)	Database • GIS
Factor Type(s)	Regulatory documents
Sample Characteristic - Organism	Vitis vinifera
Sample Characteristic - Environment	agriculture
Sample Characteristic - Location	Europe

## Background & Summary

The Protected Designation of Origin (PDO) label is a European Union (EU) quality scheme that protects products made within closely defined areas, under specific physical and biological conditions and using strictly defined production practices^1,2^. In the overall list of products that are registered and protected by the EU, wine plays a major role and includes the largest share of recognized PDO (65%)^3^. The 1177 European wine PDO comprise 21 countries and a broad range of wine products, including still, sparkling, and liquor wine. As such, PDO viticulture and winemaking represent a key socioeconomic activity^4^, for instance, in 2018 more than 81 million hl of PDO wine were produced, with an export business value of around 9 billion €^5^.

The quality scheme for PDO wines was set up to protect the unique characteristics of specific wine products and to promote their high quality^6^. It includes strict regulations regarding cultivation and production processes together with the definition of the area where the grapes must be cultivated. For example, a PDO regulation may require that wines are exclusively produced from traditional vine cultivars of a region, or that they are aged for a certain amount of time in wooden barrels. To be labelled as a PDO product, a wine needs to be formally recognized by the European commission, which requires applicants to establish a direct link between the quality attributes of the product and its geographical origin^7^. In this process, the producers need to elaborate a detailed documentation that specifies the production requirements of each wine product, i.e., the product specification, and summarize it in a stand-alone report, the so-called single document. Once a wine product is recognized and registered as a PDO, the product specification along with the single document can only be amended after presenting specific reasons why the changes are required^8^. The documents produced during the application and all eventual amendments are published online in the official EU indication register eAmbrosia, that represents the legal repository of all the geographical indications for agri-food production, wine and spirits registered and protected in the EU^9,10^.

Because of the strong relation between PDO wines and the specific conditions and production practices defined during the application process, these products are highly vulnerable to any changes in the climatic, environmental, or economic conditions in the production area^1,11^. For example, warmer climate conditions are already affecting the growing suitability of several cultivars, posing significant challenges to many labelled wine products from PDO regions^12–16^. Moreover, the introduction of alien pest species from other wine growing areas is endangering the health of vines, requiring vineyard managers to use new agricultural practices for pest control^17–19^. Economic and social preferences, on the other hand, are pushing for a more sustainable management of vineyards encouraging many European winegrowers to adopt new production practices, such as organic viticulture^20–22^. All these factors are impacting wine PDO throughout Europe and are often in conflict with the regulations defined in the application documents. For instance, to maintain their quality standards, PDO areas may need to use new production practices that are different from those specified in the regulatory documents. For this reason, there is a need to thoroughly plan and develop specific adaptation strategies that consider the local conditions and legal regulations of single PDO^1,23^. However, such strategies require knowledge about the legal specifications that characterize each PDO, which is currently only available in the regulatory documents of each wine PDO and not as a harmonized dataset.

Here, we present the first geospatial inventory of regulatory information for all 1177 PDO areas across Europe (as of 04.11.2021). We collected, standardized and spatialized a set of regulatory information from the EU indications register eAmbrosia and aggregated it in a harmonized dataset. This information is intended to be a fundamental support to inform research and decision making in the field of viticulture. For instance, crop modellers can use the information to model possible scenarios of climate impacts and adaptation in wine PDO areas. Agronomists can suggest new wine growing strategies by comparing information about different PDO, and decision makers can plan possible actions to improve high quality grapevine production.

## Methods

**Fig. 1:**
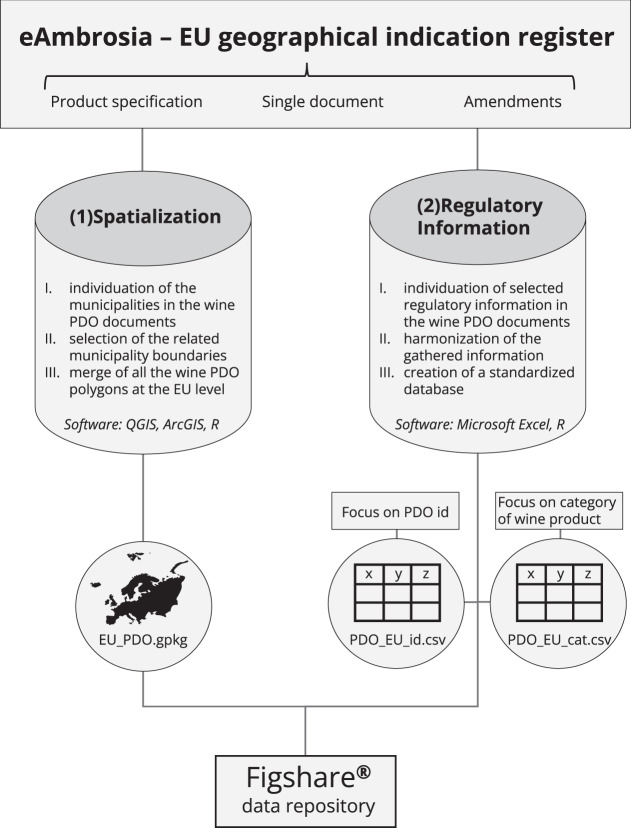
Conceptual diagram of the methodology and data formats used to build the inventory. (1) Spatialization: mapping of the municipalities included in the wine PDOs, creation of a .gpkg spatial dataset. (2) Regulatory information: extraction and harmonization of selected regulatory information, creation of two different .csv datasets one with a focus on the PDO level and the other with a focus on the categories of wine products in each PDO.

Extraction and standardization of the information from the legal documents was carried out during the period March 2021 – November 2021 using the EU geographical indication register eAmbrosia as a source^3^. The last time we checked for any changes to the legal documents of the PDO regions was on 04.11.2021; PDO areas or amendments that were published after this date are therefore not included in the present dataset. We focused on all PDO recognized in the EU and the United Kingdom. The two main steps of the process were: (1) the spatialization of the wine PDO cultivation areas and (2) the selection and standardization of regulatory information for each PDO (Fig. 1). Team members were fluent in Italian, French, German, Spanish, Portuguese, and English. The knowledge of these languages was helpful, because in most cases the regulatory documents are provided in the language of the country where the PDO is located and 80% of European wine PDO are located in Italy, France, Germany, Austria, Spain and Portugal. In case the team was not fluent in the language of a document, we had to use Regulation (EU) No 1308/2013^10^ and the commission implementing regulation (EU) 2019/33^24^ and 34^9^. These documents specify the rules for PDO regulation in the EU and the guidelines to write the application as PDO for wine products, respectively, and are translated in all the EU languages. We used them to find relevant keywords in the different EU languages that we were not fluent with and then used these keywords to find the relevant information in the regulatory documents. This was necessary for part or all of the PDOs located in Bulgaria, Belgium, Croatia, Cyprus, Czech Republic, Greece, Hungary, Romania, Slovakia and Slovenia. If any of the official documents were not available in the eAmbrosia register, we searched on dedicated websites for each country or contacted the related governmental institution to obtain the missing information.

### STEP 1: spatialization of PDO cultivation areas

**Fig. 2:**
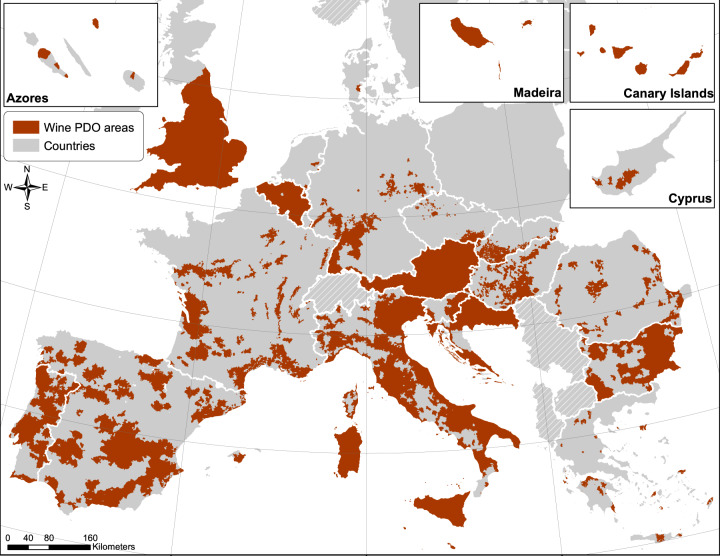
Overview of the area covered by the 1177 PDO included in the inventory. Non-European countries are represented by the striped pattern, we included United Kingdom as it was part of the EU until recently (© EuroGeographics for the country boundaries^38^).

In the vast majority of legal documents, the PDO area was defined by including a list of municipalities where the cultivation of grapes for the PDO wines is allowed. For this reason, we georeferenced the wine PDO areas using the administrative boundaries at the municipal scale provided by the EuroRegionalMap dataset (© EuroGeographics 2022^25^) as the minimum mapping unit. For each PDO, we copied the municipality names from the legal document one-by-one, manually extracted the corresponding boundaries in the geographic dataset, merged all the single municipalities and finally exported the PDO boundary as a single shapefile. For countries where the EuroRegionalMap did not include information on the municipality boundaries, we used administrative boundaries from other repositories^26,27^. This was necessary for Bulgaria, Hungary, Slovenia, Romania, Denmark, United Kingdom and Greece. In some cases, the documentation provided a detailed outline of the boundaries of the PDO area but did not include any reference to the municipality, typically only indicating specific landscape features such as roads and rivers. In these cases, we manually selected the relevant municipalities using satellite images from various sources (e.g., Esri^28^, Google Earth^29^) as a reference. Once we obtained the municipal polygons that together constitute the total PDO cultivation area, we dissolved them first to have a single polygon per PDO and then merged each single PDO together to build a unified spatial dataset (Fig. 2). The steps to spatialize the PDO areas were carried out using the QGIS^30^, ArcGIS^31^ and R^32^ software.

### STEP 2: extraction of PDO regulatory information

In the second step, we extracted a set of regulatory information from the official documents in the eAmbrosia portal. The available information was heterogenous between the single EU countries, with some of them providing very detailed information while others provided only very little information. We collected only regulatory information that was available for all included countries and could be standardized among all PDO areas. Therefore, we had to exclude a set of information such as the training system, chemical composition of wines (e.g., sugars and acid contents), organoleptic profiles and alcoholic strength. For some PDO areas, mostly those located in Italy and France, we also found that more detailed regulations regarding planting densities or yields are available, that are specified depending on varieties, wine product or even topographic conditions (e.g., localization in steep slopes). We aggregated information from more detailed regulations to the same level of detail as in the other countries. The selected regulatory information that we extracted is presented in Table 1, including the methodology that was used for standardization. To extract the regulatory information from the legal documents and insert them into our dataset, we copied the relevant entries and pasted them in a dedicated spreadsheet table before proceeding with their standardization, using the Excel software^33^.

**Table 1:** Regulatory information included in our inventory dataset.

Regulatory information	Method
Country name (*Country*)	The ISO 3166-1 code of the country where the wine PDO is located.
PDO identifier (*PDOid*)	The official id of the wine PDO as defined in eAmbrosia.
PDO name (*PDOnam*)	The official name of the wine PDO as defined in eAmbrosia.
PDO registration date (*Registration*)	The date of registration of the wine PDO.
Category of wine product (*Category_of_wine_product*)	The wine product categories allowed in each PDO, following the definition of Regulation (EU) No 1308/2013.
Vine varieties (*Varieties_OIV*)	The list of the vine varieties allowed in the wine PDO, using the nomenclature adopted by the International Organization of Vine and Wine (OIV)^37^.
Vine varieties (*Varieties_Other*)	The list of vine varieties allowed in the wine PDO that are not included in the OIV list.
Yield (*Maximum_yield_hl*)	The maximum yield allowed in the PDO areas expressed in hl/ha.
Yield (*Maximum_yield_kg*)	The maximum yield allowed in the PDO areas expressed in kg/ha.
Planting density (*Minimum_planting_density*)	The minimum planting density allowed in a PDO, expressed in number of vine stocks/ha.
Irrigation (*Irrigation*)	The extent to which it is possible to use irrigation in the PDO. Possible values are:
	• “allowed”, if irrigation is allowed. This includes the cases in which irrigation is: (i) allowed in all situations; (ii) allowed upon request to a specific regulatory organization; (iii) allowed only in emergency situations;
	• “prohibited”, if irrigation is prohibited in any cases;
	• “na”, if no information about irrigation is provided in the documents.
Presence of amendments (*Amendment*)	The presence or absence (Yes/No) of changes in the original application documents of the PDO. We considered an amendment only when a justification of the changes was provided.
General information on the PDO (*PDOinfo*)	The link to the eAmbrosia page that include the regulatory documents about a wine PDO.
Municipalities included in the PDO (*Municip_nam*)	The name of the municipalities included in the wine PDO.
Date of final check for changes in the legal documents of the PDO *(begin_lifes)*	The date when we last checked the eAmbrosia database for possible changes in the legal documents. In our case, this corresponds to 04.11.2021.

Each row corresponds to a unique field in the regulatory information dataset (the name of the field is indicated in brackets). The table includes the methodology used to standardize the information.

Once all the regulatory information for the European wine PDOs was gathered, we aggregated them either based on their PDO identifier (*PDOid* field) or based on the wine product information (*Category_of_wine_product* field). This was necessary because we wanted to provide both, a dataset that gives an overview of the wine PDOs in Europe and their main characteristics, and a dataset that is dedicated to wine products. Therefore, two distinct files were compiled:

I. A dataset containing 1177 entries, one for each PDO. It gives an overview of the main regulatory information, including the maximum yield, the minimum planting density and a list field with all the authorized vine varieties in a PDO. All the data regarding the remaining regulatory information are included in full detail.
II. A dataset containing 1983 entries, with a focus on the wine products of each PDO. It includes information about maximum yield and minimum planting density per category of wine product that is produced in each PDO. The authorized vine varieties for each category of wine product are specified in a list field. All the data regarding the remaining regulatory information are fully included.

**Fig. 3:**
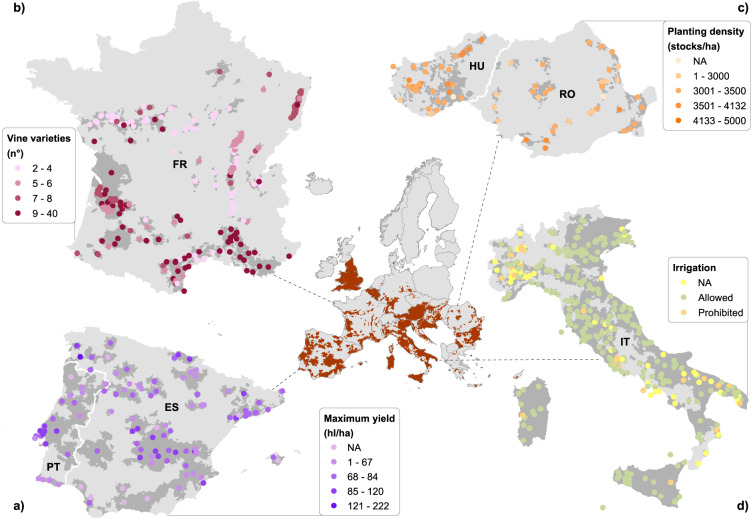
Selection of key variables included in our inventory for different countries. (**a**) Maximum allowed yield, (**b**) number of vine varieties per PDO, (**c**) minimum allowed planting density, (**d**) possibility to use irrigation. PDO areas are represented in red in the overview map and in dark grey in the inset maps. The points in the maps represent the centroids of the PDO regions.

Table 2 summarizes some of the information gathered in STEP 1 and STEP 2. Figure 3 represents a selection of key variables included in our inventory for different countries.

**Table 2:** Summary of some characteristics gathered in the geospatial inventory, aggregated at the country level. PDO (n°): total number of PDOs per country; Municipalities (n°): number of municipalities within PDO regions per country; PDO area (km²): area of the municipalities within PDO regions per country; Cultivated varieties (n°): number of varieties allowed for cultivation in the PDOs of a country; Wine products (n°): number of wine products that can be produced in the PDOs of the respective country; CLC vineyards included in PDO boundary [%]: the percentage of vineyards from the Corine Land Cover^35^ that is included in the PDO area, the “−” symbol indicates countries for which no vineyard was present in the Corine Land Cover dataset. Percentages have been rounded.

Country	PDO (n°)	Municipalities (n°)	PDO area (km^2^)	Cultivated varieties (n°)	Wine products (n°)	CLC vineyards included in PDO boundary [%]
Austria	24	2096	83930	40	2	100%
Belgium	7	570	31232	47	3	—
Bulgaria	52	156	69770	49	2	89%
Croatia	18	515	47816	154	5	100%
Cyprus	7	78	970	24	3	44%
Czech Republic	11	380	5840	69	11	100%
Denmark	1	1	605	8	1	—
France	361	4999	88496	163	7	91%
Germany	19	1473	37631	142	5	100%
United Kingdom	3	348	178271	83	2	—
Greece	33	502	8746	39	5	43%
Hungary	33	621	25748	84	5	98%
Italy	408	4923	209706	514	9	100%
Luxembourg	1	12	245	15	2	100%
Malta	2	68	313	31	4	—
Netherlands	6	18	1499	25	8	—
Portugal	30	2101	55427	237	5	95%
Romania	40	339	25510	68	4	56%
Slovakia	8	702	12562	44	8	97%
Slovenia	14	123	10285	51	2	100%
Spain	99	2858	174734	167	10	98%

## Data Records

We present an easily accessible and freely available inventory for the current wine PDO areas in the EU, comprising geospatial information as well as a set of regulatory information that can be used by researchers and decision makers. The data is freely available through the Figshare data publisher^34^.

It includes a geospatial file that contains the boundaries of the analysed PDO and two .csv files that contain the regulatory information aggregated either based on the PDO identifier or based on the category of wine product. The .csv files were saved using commas (“,”) to separate the columns, and a point (“.”) as decimal separator. Both .csv files are saved using utf-8 encoding. The files included in the inventory are:

1. EU_PDO.gpkg: a geopackage file that includes the boundaries for each of the 1177 PDO areas defined in the regulatory documents from eAmbrosia. The join between the spatial features and the other files is guaranteed by the common field *PDOid*.
2. PDO_EU_id.csv: a .csv file that includes the regulatory information outlined in Table 1 aggregated based on the PDO (*PDOid* field).
3. PDO_EU_cat.csv: a .csv file that includes the regulatory information outlined in Table 1 aggregated based on the category of wine product (*Category_of_wine_product* field).

## Technical Validation

We spatialized and gathered the regulatory information about wine PDO in Europe based on the official geographical indication register eAmbrosia, that constitutes the legal basis for PDO designation in the EU. In many cases, more than 90% of all the vineyards identified by the Corine Land Cover^35^ map are also included in the spatialized wine PDO areas (Table 2). For each PDO, we provide the reference to the official documents from which the data was created, allowing the user to cross check pieces of information with ease. Throughout the spatialization of the PDO and the collection of related regulatory information, spot checks were conducted at various stages of the progress to verify that mistakes had been kept to a minimum.

## Usage Notes

Given the amount of information included in our inventory and its coverage, this dataset will be particularly useful for researchers and decision makers in the field of viticulture. For example, the knowledge of regulatory information, such as the planting density, yield, and vine variety, can be used by researchers to calibrate crop models and generate projections of phenology and water stress indicators in PDO areas^12^. The results of these models can be compared to the characteristics of the authorized vine cultivars planted in a PDO to develop adaptation solutions for climate change such as the inclusion of new vine cultivars or the authorization of irrigation in the regulatory documents^19^. Analysing the contents of PDO documents and the related amendments can also improve our understanding of the critical factors that determine the sustainability and reputation of PDO regions. For example, Marescotti *et al*.^7^, studied the amendments of protected geographical indications in the fruit and vegetable sector and found that there is a strong need to introduce more environmental criteria in the regulatory documents, and Scozzafava *et al*.^36^, analysed how a change in PDO regulations can promote the premium products from a wine PDO region.

## Data Availability

No custom code has been used during the generation and processing of this dataset.

## References

[1] Clark LF, Kerr WA (2017). Climate change and terroir: The challenge of adapting geographical indications. J. World Intellect. Prop..

[2] European Commission - Food, Farming, Fisheries. *Quality schemes explained* https://ec.europa.eu/info/food-farming-fisheries/food-safety-and-quality/certification/quality-labels/quality-schemes-explained_en (2022).

[3] European Commission - Food, Farming, Fisheries. *eAmbrosia, the EU geographical indication register* https://ec.europa.eu/info/food-farming-fisheries/food-safety-and-quality/certification/quality-labels/geographical-indications-register/ (2022).

[4] European Commission – Directorate - General for Agriculture and Rural Development. *Study on economic value of EU quality schemes, geographical indications (GIs) and traditional specialities guaranteed (TSGs): final report* (Publications Office of the European Union, 2021).

[5] European commission - Food, Farming, Fisheries. *Wine market observatory* https://ec.europa.eu/info/food-farming-fisheries/farming/facts-and-figures/markets/overviews/market-observatories/wine_en#:~:text=The%20aim%20of%20the%20EU,analysis%20in%20a%20timely%20manner (2022).

[6] Zappalaglio A (2019). The Debate Between the European Parliament and the Commission on the Definition of Protected Designation of Origin: Why the Parliament Is Right. IIc – Int. Rev. Intellect. Prop. Compet. Law.

[7] Marescotti A (2020). Are Protected Geographical Indications Evolving Due to Environmentally Related Justifications? An Analysis of Amendments in the Fruit and Vegetable Sector in the European Union. Sustainability.

[8] Ruiz XFQ (2018). How are food Geographical Indications evolving? – An analysis of EU GI amendments. *Brit*. Food J.

[9] European commission *Commission implementing regulation (EU) 2019/34 - of 17 October 2018 laying down rules for the application of Regulation (EU) No 1308/2013 of the European Parliament and of the Council as regards applications for protection of designations of origin, geographical indications and traditional terms in the wine sector, the objection procedure, amendments to product specifications, the register of protected names, cancellation of protection and use of symbols, and of Regulation (EU) No 1306/2013 of the European Parliament and of the Council as regards an appropriate system of checks* (Official journal of the European Union, 2019).

[10] European commission *Regulation (EU) No 1308/2013 of the European Parliament and of the Council of 17 December 2013 establishing a common organisation of the markets in agricultural products and repealing Council Regulations (EEC) No 922/72, (EEC) No 234/79, (EC) No 1037/2001 and (EC) No 1234/2007* (Official journal of the European Union, 2013).

[11] International Organization of Vine and Wine. *Resolution OIV/VITI 333/2010* https://www.oiv.int/public/medias/379/viti-2010-1-en.pdf (2010).

[12] Fraga H, Atauri IG, de C, Malheiro AC, Santos JA (2016). Modelling climate change impacts on viticultural yield, phenology and stress conditions in Europe. Global Change Biol..

[13] Neethling E, Barbeau G, Coulon-Leroy C, Quénol H (2019). Spatial complexity and temporal dynamics in viticulture: A review of climate-driven scales. Agr. Forest Meteorol..

[14] Hannah L (2013). Climate change, wine, and conservation. Proc. National Acad. Sci..

[15] Tscholl, S., Tasser, E., Tappeiner, U. & Vigl, L. E. Coupling solar radiation and cloud cover data for enhanced temperature predictions over topographically complex mountain terrain. *Int. J. Climatol* (2021).

[16] Fraga H, Molitor D, Leolini L, Santos JA (2020). What Is the Impact of Heatwaves on European Viticulture? A Modelling Assessment. Appl. Sci..

[17] Daane K, Vincent C, Isaacs R, Ioratti C (2018). Entomological Opportunities and Challenges for Sustainable Viticulture in a Global Market. Annual Review of Entomology.

[18] Caffarra A, Rinaldi M, Eccel E, Rossi V, Pertot I (2012). Modelling the impact of climate change on the interaction between grapevine and its pests and pathogens: European grapevine moth and powdery mildew. Agric. Ecosyst. Environ..

[19] Santos JA (2020). A Review of the Potential Climate Change Impacts and Adaptation Options for European Viticulture. Appl. Sci..

[20] International Organization of Vine and Wine. *Focus OIV, the world organic vineyard* https://www.oiv.int/public/medias/8514/en-focus-the-world-organic-vineyard.pdf (2021).

[21] Strub L, Kurth A, Loose SM (2020). Effects of Viticultural Mechanization on Working Time Requirements and Production Costs. *Am*. J. Enol. Viticult..

[22] Marín D (2021). Challenges of viticulture adaptation to global change: tackling the issue from the roots. Aust. J. Grape Wine R..

[23] Neethling E, Petitjean T, Quénol H, Barbeau G (2017). Assessing local climate vulnerability and winegrowers’ adaptive processes in the context of climate change. Mitig. Adapt. Strat. Gl..

[24] European *commission Commission delegated regulation (EU) 2019/ 33 of 17 October 2018 supplementing Regulation (EU) No 1308/2013 of the European Parliament and of the Council as regards applications for protection of designations of origin, geographical indications and traditional terms in the wine sector, the objection procedure, restrictions of use, amendments to product specifications, cancellation of protection, and labelling and presentation* (Official journal of the European Union, 2019).

[25] Eurogeographic. EuroRegionalMap. https://www.mapsforeurope.org/datasets/euro-regional-map (2022).

[26] United Nation Office for the Coordination of Humanitarian Affairs. The Humanitarian Data Exchange. https://data.humdata.org/ (2022).

[27] Geoadata.gov.gr. Boundaries of the local authorities. https://geodata.gov.gr/en/dataset/oria-ota-pro-kapodistria/resource/5434dd9f-2b8c-4a03-84f4-63e3803c3e41 (2022).

[28] Environmental Systems Research Institute (ESRI). World street map. *Sources: Esri, DeLorme, HERE, USGS, Intermap, iPC, NRCAN, Esri Japan, METI, Esri China (Hong Kong), Esri (Thailand), MapmyIndia, Tomtom* (2022).

[29] Google. Google Earth Pro: release 7.3.4.8248 https://www.google.com/intl/en-GB/earth/ (2022).

[30] QGIS Development Team. QGIS Geographic Information System, version 3.22.4. *Open Source Geospatial Foundation Project* https://qgis.org/en/site/ (2016).

[31] ArcGIS desktop, version 10.8. *Environmental Systems Research Institute (ESRI)* (2022).

[32] R core team. R: A language and environment for statistical computing, version 4.1.2. *R Foundation for Statistical Computing* https://www.R-project.org/ (2022).

[33] Microsoft Corporation. Microsoft Excel. https://office.microsoft.com/excel (2018)

[34] Candiago S, Tscholl S, Bassani L, Fraga H, Egarter Vigl LA (2022). figshare.

[35] European Environment Agency (EEA). Corine land cover 2018 version 2020. *Copernicus programme* https://land.copernicus.eu/pan-european/corine-land-cover/clc2018 (2022).

[36] Scozzafava G, Gerini F, Dominici A, Contini C, Casini C (2018). Reach for the stars: The impact on consumer preferences of introducing a new top-tier typology into a PDO wine. Wine Econ. Policy.

[37] International Organization of Vine and Wine. *International list of vine varieties and their synonyms* https://www.oiv.int/en/technical-standards-and-documents/description-of-grape-varieties/international-list-of-vine-varieties-and-their-synonyms (2013).

[38] Eurostat, GISCO: Geographical Information and Maps. LAU 2020. © EuroGeographics for the administrative boundaries https://ec.europa.eu/eurostat/web/gisco/geodata/reference-data/administrative-units-statistical-units/lau (2022).

